# The metastasis suppressor RARRES3 as an endogenous inhibitor of the immunoproteasome expression in breast cancer cells

**DOI:** 10.1038/srep39873

**Published:** 2017-01-04

**Authors:** Alison M. Anderson, Murugan Kalimutho, Sarah Harten, Devathri M. Nanayakkara, Kum Kum Khanna, Mark A. Ragan

**Affiliations:** 1 Institute for Molecular Bioscience, The University of Queensland, Brisbane QLD 4072, Australia; 2 Signal Transduction Laboratory, QIMR Berghofer Medical Research Institute, 300 Herston Road, Herston, Brisbane QLD 4006, Australia

## Abstract

In breast cancer metastasis, the dynamic continuum involving pro- and anti-inflammatory regulators can become compromised. Over 600 genes have been implicated in metastasis to bone, lung or brain but how these genes might contribute to perturbation of immune function is poorly understood. To gain insight, we adopted a gene co-expression network approach that draws on the functional parallels between naturally occurring bone marrow-derived mesenchymal stem cells (BM-MSCs) and cancer stem cells (CSCs). Our network analyses indicate a key role for metastasis suppressor *RARRES3*, including potential to regulate the immunoproteasome (IP), a specialized proteasome induced under inflammatory conditions. Knockdown of RARRES3 in near-normal mammary epithelial and breast cancer cell lines increases overall transcript and protein levels of the IP subunits, but not of their constitutively expressed counterparts. *RARRES3* mRNA expression is controlled by interferon regulatory factor *IRF1,* an inducer of the IP, and is sensitive to depletion of the retinoid-related receptor *RORA* that regulates various physiological processes including immunity through modulation of gene expression. Collectively, these findings identify a novel regulatory role for *RARRES3* as an endogenous inhibitor of IP expression, and contribute to our evolving understanding of potential pathways underlying breast cancer driven immune modulation.

Aberrant epithelial-mesenchymal transition (EMT) is thought to confer subpopulations of cancer cells, commonly referred to as cancer stem cells (CSCs), with stem-like properties that enhance capacity to disseminate, migrate and initiate tumors at distal sites^1^. The activity of CSCs is coupled with compromise or dysfunction of the immune system involving subversion of the dynamic continuum involving pro- and anti-inflammatory regulators^2^,^3^. While much progress has been made in understanding pathological EMT^1^, the immune-modulatory mechanisms of CSCs remain poorly understood. In addition to stem-like properties, CSCs display characteristics similar to those of naturally occurring bone marrow-derived mesenchymal stem cells (BM-MSCs)^4^,^5^,^6^,^7^,^8^, and appear to ‘hijack’ signaling circuits used by these cells during wound repair including chemokine-dependent migration and homing to distance sites as well as immune modulation^9^,^10^. For instance, the chemokine ligand *CCL18* is responsible for the induction of BM-MSC-induced immune suppressive *FOXP3*^+^ regulatory T cells^11^, and in a murine model of breast cancer, a positive feedback loop between CSCs and macrophages expressing *CCL18* has been shown to drive metastasis^12^.

When grown as single-cell-derived progenies and inoculated into mice, cells from the human breast cancer cell line MDA-MB-231 display distinct metastatic abilities and tissue tropisms^13^,^14^,^15^. Expression profiling of these progenies has provided gene signatures whose expression pattern associates with metastasis to brain^13^, bone^14^ and/or lung^15^. Most of the genes identified encode secreted and extra-cellular products including growth and survival factors, chemokines and cell surface proteins, indicating that the metastatic process may involve mechanisms beyond those underpinning the initiation and growth of the primary tumour^14^,^15^. We envisaged that these metastasis-specific gene signatures might hold important clues in regards to immune-modulatory processes that are increasingly understood to play critical roles during metastasis^2^.

Gene-enrichment approaches that map gene signatures of interest to experimentally supported knowledge resources including the Gene Ontology (GO) and Ingenuity Pathway Analysis (IPA) are biased to current knowledge, and often link genes to multiple and/or broad functional terms, making it difficult to discern specific mechanisms or critical interactions. Furthermore, the metastatic gene signatures represent differentially expressed genes, and it is likely that important regulators that exhibit minimal or no change in mRNA levels across the conditions tested, are also in play.

In the current study we adopted an alternative network-based approach that draws on the functional parallels between BM-MSCs and CSCs. We construct a gene set to represent the biology of interest which includes common breast cancer markers, chemokines, and cell surface markers generally used to characterise BM-MSCs. Using this gene set, and microarray data obtained from BM-MSCs in which an immune response had been reported, we generate a gene co-expression network. Overlay on the network of genes from the metastatic gene signatures reveals a 177-gene module (hereinafter referred to as the *metastatic module)*, providing insight into immune response signaling likely perturbed during breast cancer metastasis. Co-expression patterns indicate a role for the retinoic acid receptor responder protein 3 (*RARRES3*). This gene encodes a multi-functional enzyme associated with varied metastasis suppressing mechanisms including intrinsic phospholipase A1/A2 activity^16^ and palmitoylation of Wnt/β-catenin signaling molecules^17^.

Correlated expression between *RARRES3* and immunoproteasome (IP) subunits (*PSMB8, PSMB9* and *PSMB10*) observed within a BM-MSC network are conserved across datasets representing breast cancer cells and tissue. The subunit *PSMB8* is implicated in autoimmune disease^18^, thus an improved understanding of IP regulatory mechanisms could provide useful insights into breast cancer-induced immune dysregulation.

Knockdown, and overexpression, of *RARRES3* in near-normal mammary epithelial cells and breast cancer cell lines confirm a capacity of the retinoid-responder gene to modulate the IP catalytic subunits both at transcriptional and protein levels, suggesting a novel regulatory role of RARRES3 in breast cancer. This activity is controlled by depletion of the interferon regulatory factor (*IRF1*), a known inducer of the IP subunits and the retinoic acid receptor-related orphan receptor alpha (*RORA*).

## Results

### Construction of a BM-MSC co-expression network

BM-MSC data were obtained from a study in which the authors reported an unexpected immunological effect mediated by retinoic acid-inducible gene 1 (*RIG-1/DDX58*)^19^. The extracted gene expression profiles are thus likely to represent the immunomodulatory function (as opposed to the stem/progenitor properties of BM-MSCs), and as such, were considered appropriate for elucidating gene modules that might be involved in immunomodulation-related events. Furthermore, *DDX58* has been identified in several breast cancer related studies^20^,^21^, and is a component of a 21-gene interferon signature with subtype-dependent prognostic value in lymph node-negative patients^22^.

Given our hypothesis that breast cancer stem cells mimic BM-MSCs, we chose to seed the network using a gene set representing key characteristics of these cell types, and the chemokine signaling pathways they mediate (described in Methods) (Supplementary Table 1). We computed the Pearson correlation coefficient (PCC) between each gene in this candidate gene set and each other available on the microarray platform, to construct a network in which nodes represent genes and the edges represent the PCC between the expression of connected genes (cut-off at *r* > = |0.40|, p < 0.05 after adjustment for multiple testing using Benjamini Hochberg correction).

**Figure 1:**
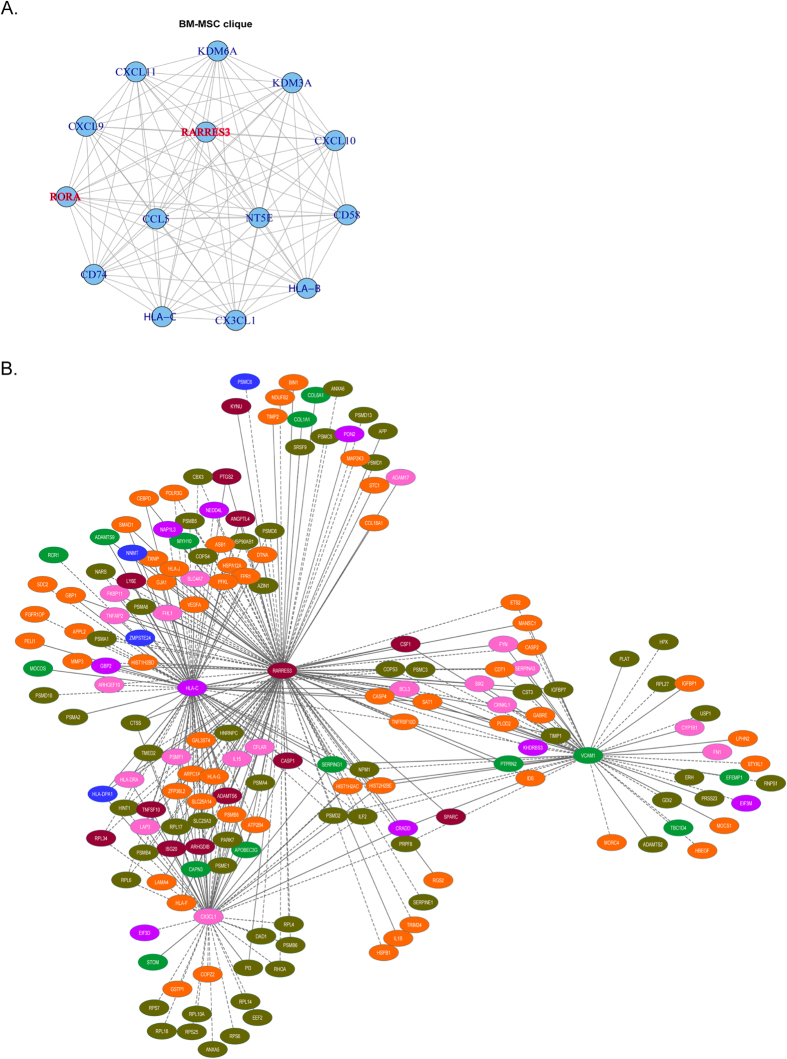
(**A**) BM-MSC 14-gene clique. A clique is a sub-network in which each gene is connected to all others (PCC > |0.40| adjusted *p* < 0.05). Cliques can identify genes that are associated with a specific biological function. A clique comprising 14 genes was identified using the *largest.clique* function available within the R *igraph* library. (**B**) A177**-**gene module comprising genes implicated in breast cancer metastasis to bone, lung and/or brain. The co-expression network was derived from BM-MSCs (PCC > |0.40|, adjusted p-value < 0.05). Node color indicates the site of metastasis the gene has been associated with. Pink = bone, green = lung, orange = brain, mauve = bone and brain, blue = bone and lung, brown = brain and lung and forest green = bone, brain and lung.

Analysis of the resulting co-expression network (6,586 nodes, 69,749 edges) identified a 14-gene clique (i.e. each gene is connected to all others at PCC > |0.40| adjusted *p* < *0.05*) (Fig. 1A), indicating that these genes share common biological function^23^. As expected, the clique includes cell-surface markers that are used to characterise BM-MSCs, and several chemokines. Less expected was the presence of the tumor suppressor *RARRES3*, which also had the highest degree of connectivity (number of neighbors) within the overall network (linking to 3337 of the 6586 genes). This was surprising, as to the best of our knowledge a role for this gene within BM-MSC and/or immune biology has not been previously reported. However, another retinoid-related gene, Retinoic Acid Receptor-Related Orphan Receptor alpha (*RORA*), for which a role in pro-inflammatory cytokine regulation is described^24^ also had a high degree (2,609) of connectivity and was present within the clique (Fig. 1A).

### Breast cancer metastasis genes within the BM-MSC network

Genes associated with breast cancer metastasis to bone, brain, and lung were obtained from the literature^13^,^14^,^15^,^25^ (Supplementary Table 2). From a total of 667 genes, 623 were available on the microarray, and 267 of these were identified within the BM-MSC network. Two-thirds of these formed a single gene module, hereinafter referred to as the *metastatic module* (Supplementary Table 3, Fig. 1B). The high level of correlation amongst these genes is to be expected, given that they were identified due to their simultaneous dysregulation in experimental models of metastasis. The metastatic module represents how they might interact with each other within an immune response context, and elucidates immune mechanisms that might be perturbed during the metastasis process. As with the overall BM-MSC network, *RARRES3* has the highest number of immediate neighbors, linking to 77% (136/177) of the metastatic-module genes.

### Co-expression patterns indicate a role for the immunoproteasome

**Figure 2:**
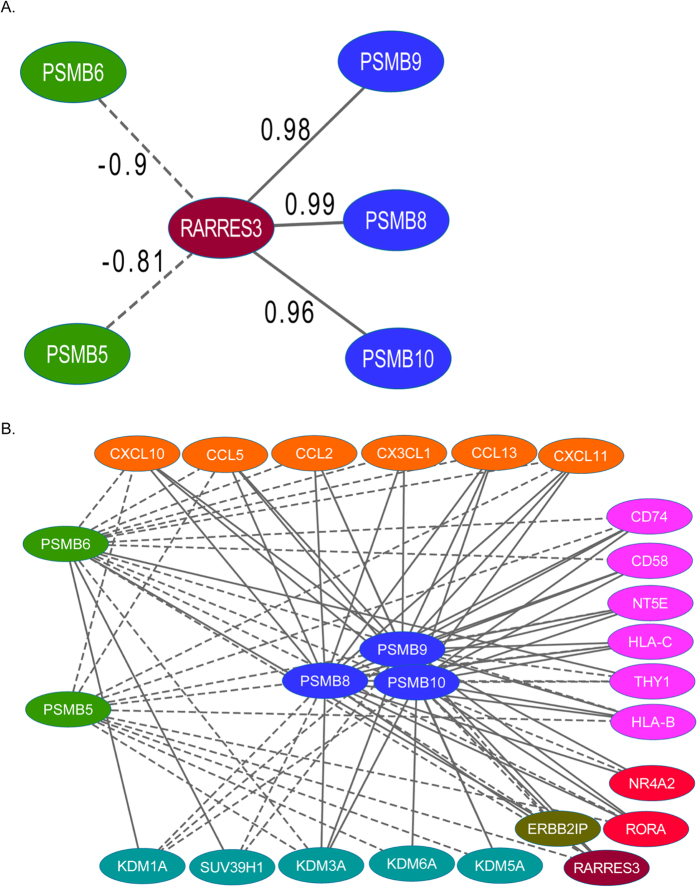
Co-expression patterns involving RARRES3 and proteasome subunits. (**A**) *RARRES3* expression is positively correlated with IP subunits and negatively correlated with CP subunits (PCC indicated on edges, adjusted p-value < 0.05). (**B**) First neighbors common to both CP and IP subunits consistently show opposing direction of correlation with these genes (PCC > |0.40|, adjusted p-value < 0.05). Solid lines represent positive and broken lines negative, co-expression between linked genes. IP subunits are shown in blue, CP subunits in green and neighbours (and where applicable, common neighbours) are coloured according to common function (orange = chemokine ligands and receptors, turquoise = chromatin modifiers, pink = BM-MSC surface markers, red = nuclear receptors).

During the immune response, the constitutive proteasome (CP) subunits (*PSMB5/PSMB6/PSMB7*) are replaced by homologous subunits (*PSMB8/PSMB9/PSMB10*) to form the immunoproteasome (IP)^26^,^27^. Within the metastatic module, *RARRES3* is positively correlated with IP subunits and negatively correlated with CP subunits (Fig. 2A). Opposing correlated expression patterns between the CP and IP, and their shared neighbors within the module, align with the concept of IP induction during immune response (Fig. 2B). Ingenuity Pathway Analysis of the metastatic module genes further supports a role for IP activity. The top-ranked canonical pathways are the *Protein Ubiquitination Pathway* (4.31E–16) and the *Antigen Presentation Pathway* (1.93E–09). Furthermore the tumor necrosis factor (*TNF*) and interferon gamma (*IFNG*), two known inducers of the IP, are also identified as upstream regulators of this network.

**Figure 3:**
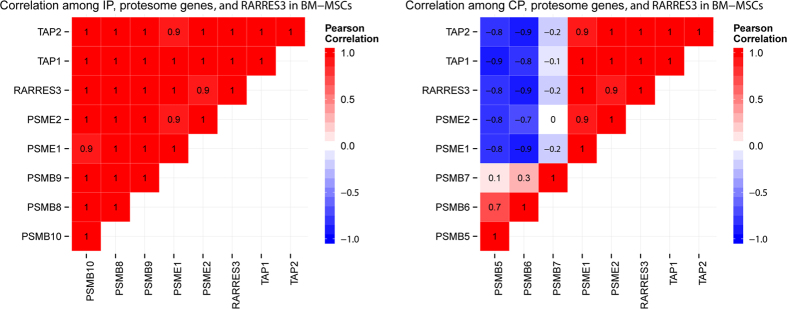
RARRES3 and IP subunits show significant positive correlation with proteasome activators and transporter genes. CP subunits *PSMB5* and *PSMB6* are negatively correlated (rounded Pearson correlation coefficients are shown on graph squares, p < 0.01), while no significant correlation is observed for *PSMB7*.

To gain further evidence of an active IP, we computed the mean Pearson correlation coefficient (PCC) between proteasome activators *PSME1* and *PSME2,* transporters for antigen processing genes *TAP1* and *TAP2,* and either the three IP or associated CP subunits and *RARRES3*. IP subunits showed positive correlation with activators and TAP genes, as did *RARRES3*, while the CP genes showed a negative correlation (Fig. 3). Given that proteasome genes are known to interact, it is expected that they would show correlated expression; the variance in mean correlation between IP and CP subunits is a possible indicator of IP induction.

### RARRES3 and IP co-expression conserved across breast cancer datasets

**Figure 4:**
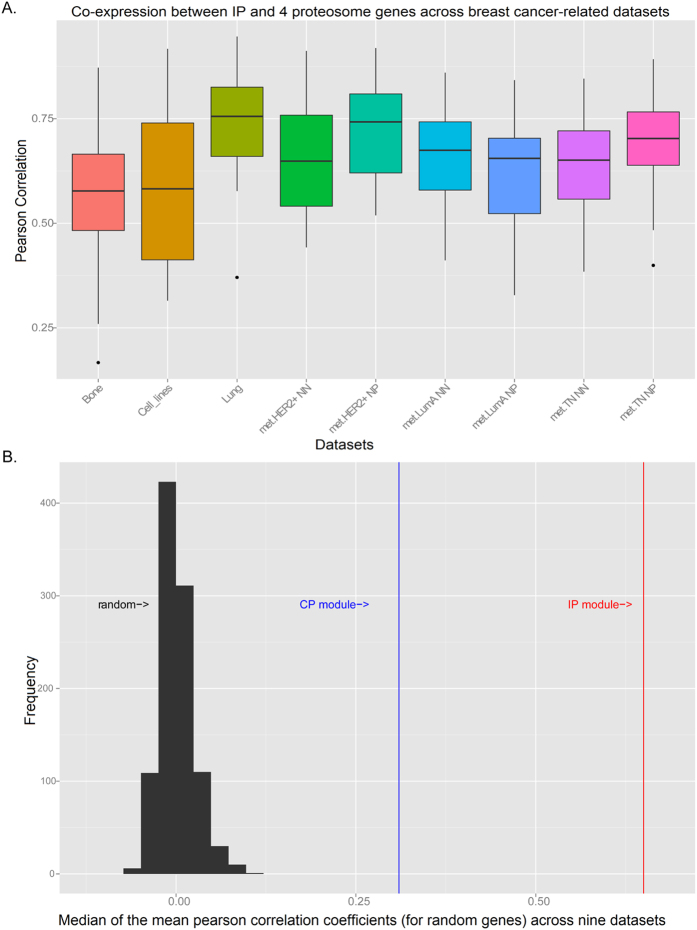
Proteasome subunit co-expression profiles across breast cancer-related datasets. (**A**) Pearson correlation coefficient (PCC)s between seven genes: three IP catalytic subunits (*PSMB8, PSMB9* and *PSMB10*), proteasome activators *PSME1* and *PSME2* and transporter genes *TAP1* and *TAP2* across cancer cells, and eight datasets representing breast cancer tumour tissue’s. (**B**) The mean PCC among seven randomly selected genes was calculated for each of nine datasets. This was conducted 1000 times. The distribution of the median mean across datasets centres around zero and is shown on the left of the graph. In comparison, the median mean PCC between CP catalytic subunits, proteasome activators (*PSME1* and *PSME2*), and TAP genes (*TAP1* and *TAP2*) across the nine datasets is 0.31 and indicated by a blue line. The red line indicates the mean PCC of the IP catalytic subunits and proteasome genes (0.65).

We next investigated the patterns of co-expression between the proteasome genes, and either the IP or CP, in data derived from breast cancer cells and tumor tissues. The latter included tumors for which the site of relapse was known to be bone or lung, as well as subtype-specific (luminal A (*ER*^+^ and/or *PR*^+^ *HER2*^*−*^), triple-negative (*ER*^*−*^ *PR*^*−*^ *HER2*^*−*^) and HER2 positive (*HER2*^+^ *ER*^*−*^ *PR*^*−*^)), lymph node status-specific (node positive (NP) and node negative (NN)) samples obtained from the METABRIC resource^28^. The median mean PCC across nine datasets was higher for the IP (0.65, 96% with *p* < 0.05) (Fig. 4A), than for the CP (median mean PCC = 0.31, 51% with *p* < 0.05, data not shown). The strength of correlation for both module compositions was greater than would be expected by chance, as determined by plotting the distribution of the median mean PCC for 1000 randomly sampled groups of genes of the same size (Fig. 4B).

As BM-MSCs have a propensity to home to tumor sites^29^, it is possible that this cell type is present within the cellular mix. Where possible, samples were restricted to those with a high cancer cellularity; nevertheless, BM-MSCs may contribute to the high correlation of expression between IP and proteasome genes in data derived from whole tissue samples. The difference in the mean of pairwise correlations (p = 0.0006) between samples from tumors associated with metastasis to bone, as compared to those that spread to lung, could indicate organ-specific mechanisms; however, this variance may be confounded by the different sample numbers available for analyses. Also, the heterogeneity of sources within the cell line dataset, which comprises 38 cell lines derived from pleural effusion and represents a range of breast cancer subtypes, may contribute to the lower overall correlation within this dataset.

**Figure 5:**
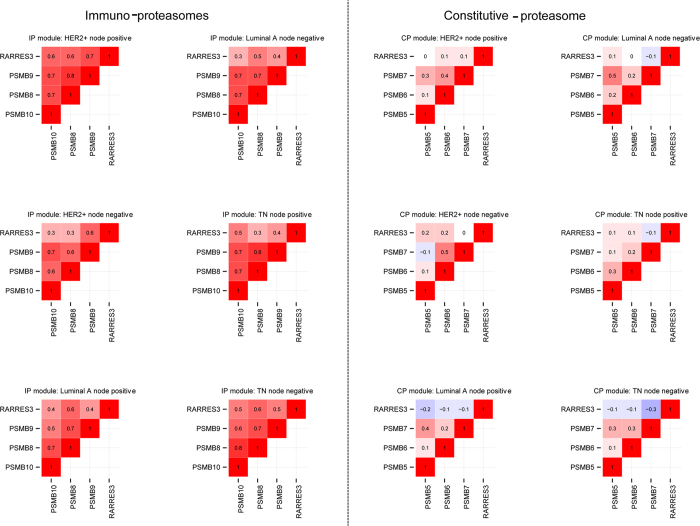
Positive correlation between RARRES3 and IP subunits, but not CP subunits, is conserved across tissue datasets obtained from the METABRIC resource. Interim analysis was conducted using different subtypes and node status networks.

A pattern of positive correlation between *RARRES3* and IP subunits was conserved across all six subtype- and lymph node status-specific datasets, while no significant correlations between *RARRES3* and the CP subunits was evident (Fig. 5). This suggests that the IP is sensitive to retinoid-related signaling and that this relationship is independent of breast cancer subtype.

### Overlap between the metastatic module and networks representing tumor tissues

**Figure 6:**
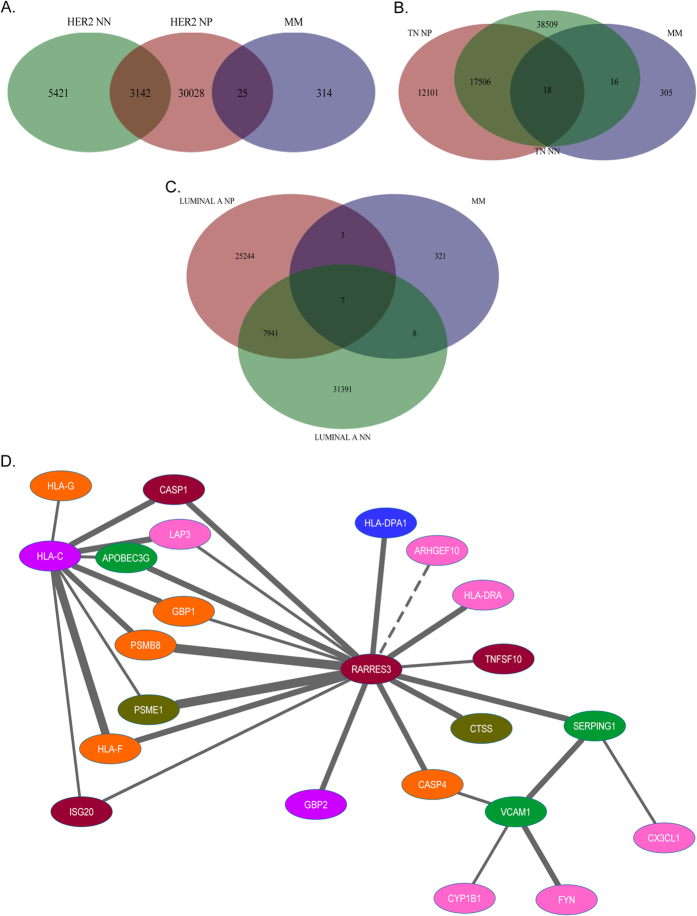
Venn diagrams show the number of correlated gene pairs that are common to the metastatic module (MM) and either the node-positive (NP), node-negative (NN), or both networks representing HER2+ (**A**), luminal A (**B**) and triple-negative (TN) (**C**) breast cancer subtypes. (**D**) 30 pairs of correlated genes were observed within multiple subtype- and node-specific networks, and link together to form a gene hub. Line thickness indicates the number of networks in which the gene pair were observed (ranging between 1 (thinnest) and 4 (thickest)). Node colour indicates the site of metastasis with which the gene has been associated: pink = bone, green = lung, orange = brain, mauve = bone and brain, blue = bone and lung, brown = brain and lung and forest green = bone, brain and lung.

To gain further insight into tissue-specific impacts we constructed co-expression networks for each of the METABRIC datasets, using the same candidate gene set and methods used to create the BM-MSC network. We then assessed the overlap between the metastatic module (MM) and each of these networks. The networks representing aberrant epidermal growth factor signaling (*HER2*^+^ and TN) had the greatest difference in overlap between lymph node negative and lymph node positive networks (Fig. 6A–C, Supplementary Table 4 and 5). In *HER2*^+^, the overlap was greatest in node positive, while in TN, the overlap was greatest in the node negative network, indicating opposing effects of *HER2* over- and under-expression.

Interestingly, 30 correlated gene pairs were observed in more than one network derived from METABRIC (Supplementary Table 6). These gene pairs link to form a 23-gene hub (Fig. 6D). This gene hub contains *RARRES3, PSMB8* and genes that encode major histocompatibility complex (MHC) class I (*HLA-C and HLA-F*) and class II molecules (*HLA-DRA and HLA-DPA1*). Proteasomes play an important role in the generation of antigen peptides for presentation on MHC molecules and the subsequent activation of CD8+ T lymphocytes^26^,^30^. We therefore considered that the relationship between *RARRES3* and *PSMB8* might provide insights into immune modulatory mechanism in breast cancer.

### Knockdown of RARRES3 up-regulates IP catalytic subunits

**Figure 7:**
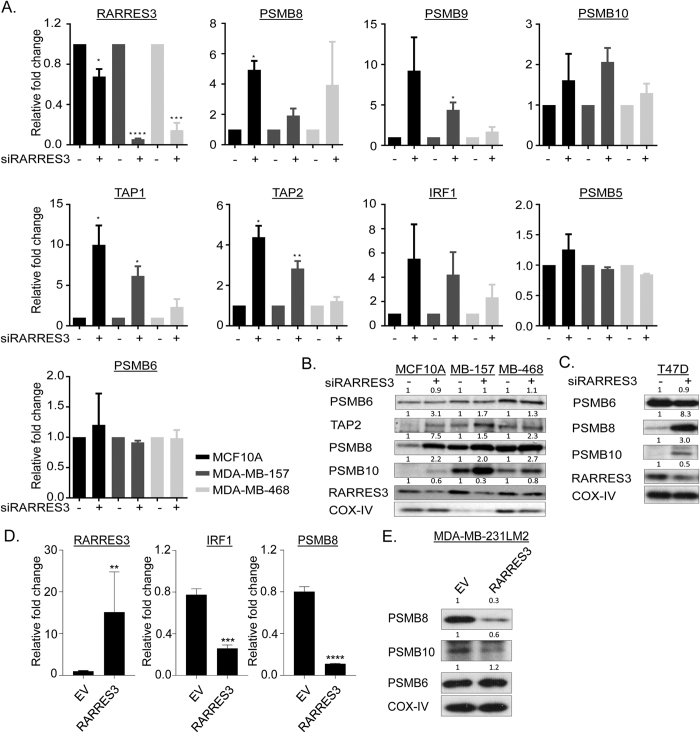
*RARRES3* knockdown modulates the catalytic subunits of IP both at mRNA and protein levels in breast cancer cell lines. (**A**) mRNA expression of genes involved in IP and CP subunits was determined following *RARRES3* knockdown in breast cancer lines. Relative fold change was calculated to scramble siRNA transfected cells after 24 hours post-transfection with 10 nM of pooled siRNA against *RARRES3*. Error bars represent the standard error of the mean from two independent experiments. (**B,C**) Immunoblot analysis with indicated antibodies was performed following *RARRES3* knockdown after 48 post transfection to determine the expression of representative IP proteins. COX-IV served as a loading control. (**D**) mRNA expression of *IRF1* and *PSMB8* following forced expression of RARRES3 in MDA-MB-231LM2 cell line and (**E**) corresponding immunoblot analysis of PSMB6, 8 and 10. COX-IV served as a loading control.

Since our network analyses indicate potential roles for *RARRES3* in regulating the IP, we investigated a functional relationship between *RARRES3* and IP catalytic subunits in selected near normal mammary epithelial cells, two *RARRES3*-expressing triple-negative cell lines and a luminal line^31^ (Supplementary Figure 1A). In support of our observation, we found that *RARRES3* messenger RNA expression was relatively down-regulated in cell lines of the basal B breast cancer subtype compared to other subtypes^31^ (Supplementary Figure 1B). Unexpectedly and surprisingly, given that the IP genes and *RARRES3* showed positive correlated expression in all our co-expression networks, knockdown of *RARRES3* resulted in up-regulation of all IP catalytic subunits across different cell lines tested, and this was verified at both RNA transcript and protein levels (Fig. 7A–C). Conversely, overexpression of *RARRES3* in the metastasis cell line MDA-MB-231LM2^16^ reduced both transcript and protein levels of IP subunits (Fig. 7D,E). The positive relationship between *RARRES3* and IP subunits in our co-expression networks derived from whole tissue samples may reflect mechanism(s) involving interaction between different cells types and/or specific cellular conditions, while our observation in near normal mammary epithelial cells and breast cancer cells may reflect mechanisms specific to these cell types. A search in the interferon database (http://www.interferome.org/) shows that up-regulation of both *RARRES3* and the IP subunits, all of which are interferon-responsive genes, occurs in only some cell types. Interestingly these include CD4^+^, CD8^+^ and natural killer cells (Supplementary Figure 2).

The TAP genes, but not the activators *PSME1* and *PSME2*, were significantly up-regulated in both MCF10A and MDA-MB-157 lines following *RARRES3* knockdown (Fig. 7A), and this was confirmed at the protein level for the TAP2 gene (Fig. 7B). The TAP genes are components of the MHC class-I-loading complex that generates antigenic peptides for presentation to CD8 T cells^26^, and the change in their mRNA levels is consistent with the conjecture that *RARRES3* activity impacts on antigen presentation pathways in breast cancer cells.

**Figure 8:**
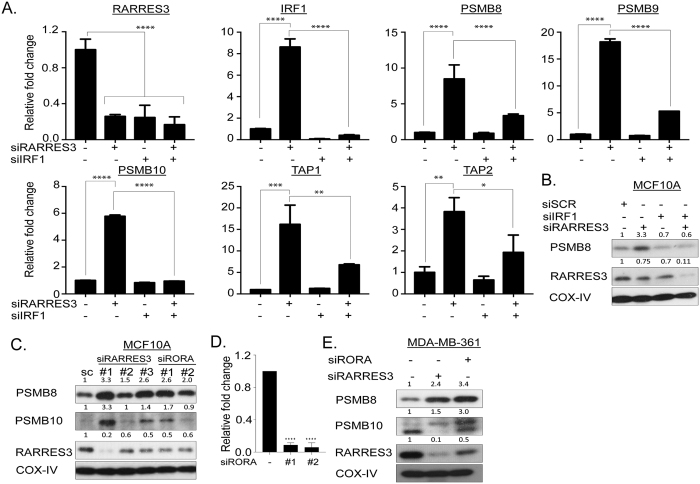
*RARRES3* modulates the catalytic subunits of IP through *IRF1* induction. (**A**) mRNA expression of genes involved in IP subunits was determined following single and combined *RARRES3* and *IRF1* knockdown in MCF10A cell line. Relative fold change was calculated to scramble siRNA transfected cells after 24 hours post-transfection with 10 nM of pooled siRNA against *RARRES3* and *IRF1*. Error bars represent the standard error of the mean from two independent experiments. (**B**) Immunoblot analysis with indicated antibodies was performed following single and combined *RARRES3* and *IRF1* knockdown. (**C,E**) Immunoblot analysis with indicated antibodies was performed following *RARRES3* or *RORA* knockdown after 48 post-transfection to determine the expression of representative IP proteins in both MCF10A and MDA-MB-361 respectively. COX-IV served as a loading control. (**D**) mRNA expression of *RORA* using two independent siRNAs to confirm the extent of depletion.

Interferon regulatory factor 1 (*IRF1)* has previously been identified as a key player in transcriptional regulation of *PSMB8*^32^, and *PSMB9*^33^ and is also sensitive to retinoic acid^34^,^35^. Here, knockdown of *RARRES3* up-regulated *IRF1* (Fig. 7A) and conversely knockdown of *IRF1* reduced *RARRES3* mRNA levels (Fig. 8A). While depletion of *IRF1 alone* had minimal to no direct effect on the expression of IP subunits or TAP genes, the combined knockdown of *RARRES3* and *IRF1* significantly reduced the increase in expression of these genes mediated by *RARRES3* depletion alone (Fig. 8A,B). Moreover, *RARRES3* expression was reduced following depletion of *RORA* while the expression of *PSMB8* and *PSMB10* were increased (Fig. 8C–E) consistent with a role for retinoid-related signalling in IP induction. Due to lack of availability of specific antibody against RORA, depletion was measured by qRT-PCR (Fig. 8D).

## Discussion

In the current study, we adopted a network approach that draws on similarities between BM-MSCs and CSCs in terms of homing to distance sites and immune modulation to identify a gene module operating at the immune axis of breast cancer metastasis. The tumour suppressor *RARRES3* emerged as a likely important mediator of immune modulation. *RARRES3* has previously been shown to attenuate breast cancer progression via lipid-related mechanisms including its intrinsic phospholipase A1/A2 activity^16^ and palmitoylation^17^. Our findings extend this functional repertoire by identifying a novel regulatory role as an endogenous inhibitor of the IP (but not the CP) subunit expression.

### RARRES3 as a regulator of IP mRNA expression

*RARRES3* regulation of the IP expression is likely complex and subject to the activity of additional co-regulators/mediators including a role for *IRF1* and *RORA* as identified in the current study. IRF1 reduces the impact of *RARRES3* knockdown on IP subunits and TAP genes, consistent with previous reports of IRF1-dependent IP induction^33^,^34^,^36^. *IRF1* regulation of target genes is specific to cell-type and cellular stimuli, one of which is retinoic acid^34^. *RORA* is commonly down-regulated in breast cancer and considered a potential tumour suppressor gene^37^. As with *RARRES3, RORA* showed correlated expression with multiple metastasis-genes in our immune-related network and knockdown of the receptor reduced *RARRES3* mRNA and protein levels in MDA-MB-361 and MCF10A cell lines. These observations suggest that retinoid-related signalling modulates IP function. The induction of the proteolytic and regulatory subunits of the IP following exposure to retinoic acid derivatives has been previously observed in neuroblastoma cells^38^ but remains poorly understood.

Further studies will be required to determine the molecular mechanisms underpinning *RARRES3* inhibition of the IP expression. RARRES3 is a member of the H-RAS-like suppressor (HRASLS) family of enzymes, all of which possess phospholipid metabolizing and acyltransferase abilities^39^. Thus it is possible that RARRES3′s modulation of the IP involves a lipid-related regulatory mechanism such as palmitoylation. Whether or not the activity of the IP subunits, or co-regulators *IRF1* and *RORA* is palmitoylation-dependent is yet to be fully investigated. Cysteine residues are the primary substrate for palmitoylation^40^. A non-catalytic cysteine, Cys48, which is exclusive to *PSMB8* is the target of a proteasome inhibitor specific to this subunit^41^ and the anti-inflammatory drug withaferin can inhibit proteasome β5 (*PSMB5/PSMB8*) irreversibly through palmitoylation of the N-terminal Thr1, which is required for the proteolytic activity of the subunits^42^. It will be important to further determine a role for palmitoylation and for *RARRES3* in IP regulation within the context of breast cancer metastasis.

### How might RARRES3-dependent IP activity contributes to immune system compromise?

A bioinformatic study conducted across seven cancer types found that gene modules representing MHC class I function are highly expressed, and that within these modules, the most up-regulated proteasome subunits are *PSMB8* and *PSMB9*^43^. Here, the expression of IP subunit *PSMB8* correlated with *RARRES3* in BM-MSC and four tumor datasets, and also correlated with HLA-C in all but one of these cancer types. Mice deficient in *PSMB8* show a reduction in MHC class I surface expression in lymphocytes^44^, and *PSMB8* null mice fail to up-regulate pro-inflammatory chemokines and cytokines including interleukin (*IL*)*-1β* after experimentally induced colitis, leading to reduced infiltration of the colon by neutrophils and reduced expansion of T helper cells^45^. This scenario has similarities with a mouse model of spontaneous breast cancer metastasis in which *IL-1β* acts as a trigger for tumor-induced neutrophil expansion, and polarization of neutrophils towards a CD8^+^ T cell-suppressive phenotype^46^. The authors of this study suggest that neutrophils promote breast cancer metastasis, and this phenomenon is evidenced at a clinical level where, for example, a low neutrophil/lymphocyte ratio is associated with favorable prognosis in triple-negative breast cancer patients who have undergone neoadjuvant chemotherapy^47^. Further investigations are required to determine whether *PSMB8* plays an early and fundamental role in pathological immune response via modulation of key pro-inflammatory mediators including *IL-1β* that, in turn, regulate dynamic ratios of pro- and anti-inflammatory immune cells.

## Concluding Remarks

We identify a novel mechanistic role for the known metastasis suppressor *RARRES3* as an endogenous inhibitor of the IP, and show that the known IP inducer *IRF1* and its associated co-regulator *RORA* mediate this activity. *PSMB8* is implicated in perturbation of the immune system in autoimmune disease and our findings indicate that its role in immune modulation within the context of breast cancer metastasis is worthy of further, focused investigation. Importantly, PSMB8-specific inhibitors should be investigated as a therapeutic option prior to onset of metastasis.

The enzyme encoded by *RARRES3* has capacity to modulate palmitoylation, a post-translational modification with strong enrichment of transmembrane proteins^48^. Given that breast cancer-metastasis gene signatures are enriched with genes that encode proteins active at the cell surface, it is interesting to speculate that aberrant *RARRES3*-dependent palmitoylation cycling may be a common factor in their dysregulation. Furthermore, the activity of many genes implicated in breast cancer are palmitoylation-dependent; however, a possible role for aberrant palmitoylation in breast cancer initiation and progression is yet to be investigated^49^. In conclusion, findings from the current study indicate that *RARRES3* and retinoic-related signaling may play a fundamental and currently underappreciated role in breast cancer-mediated immune compromise.

## Material and Methods

### Gene set

We constructed a gene set representing three main functional categories associated with our hypothesis that CSC activity during the metastasis process mirrors that of BM-MSCs during inflammatory response: nuclear receptors and receptors from the epidermal growth factor receptor family; cell-surface markers used to characterise BM-MSCs and chemokine ligands and receptors; and genes representing modification of methylation at specific histone substrates. The activity of the latter provides an indication of major cellular changes such as EMT (Supplementary Table 1).

### Tissue- and subtype-specific metastasis gene lists

Published lists of genes specific to breast cancer metastasis to bone^14^,^25^, brain^13^ and lung^15^,^25^ were used to construct a list of tissue-of-relapse-specific genes. Tissue-specific action had been determined by a variety of methods including differential expression in early stage metastasis in xenograft models and, for some genes, association with disease-free metastasis in patient cohorts. While this list is not exclusive, it captures genes implicated in the process of distal metastasis (Supplementary Table 2).

### Datasets

Datasets for construction of networks representing MSCs, cancer cells, and tumour samples for which the site of recurrence was known were all obtained from the ArrayExpress repository^50^ (Supplementary Table 7). Patient samples representing subtype-specific tumors were obtained from the Molecular Taxonomy of Breast Cancer International Consortium (METABRIC)^28^. Only METABRIC patient samples with high cellularity and for which lymph node and receptor status were available were considered for analysis (Supplementary Table 6). Datasets had either been pre-processed by the original authors, or where raw data were available, were normalized using RMA (quantile normalization) implemented in the R environment^51^ using the Bioconductor V3.1 package *affy*^52^. All data were log2-transformed, and probes with the highest median expression within probe sets were selected to represent specific genes. We chose to build networks at the gene rather than probe level as many probe group members show similar correlation profiles and thus vastly increase the number of edges in a network while adding little information. While this approach may not allow detection of important feedback loops involving different transcripts for a single gene, we reasoned that future follow-up work could involve a probe-level approach focused on important sub-networks elucidated in this initial gene-level analysis.

### Network construction and analyses

We computed the Pearson correlation coefficient (PCC) between each gene in our candidate gene set and each other available on the microarray platform for each dataset, to construct networks in which nodes represent genes and the edges represent the PCC between the expressions of connected genes. A cut-off at *r* > = |0.40| (p < 0.05 after adjustment for multiple testing using Benjamini Hochberg correction) was used. Networks were visualised using Cytoscape^53^. The R *igraph* package^54^ was used to identify clusters within large networks and Student’s *t test* was used to compare mean PCC values (obtained for two different gene modules across networks). *P* values < 0.01 were considered significant. The metastatic genes that formed a single module within the BM-MSC network were analysed using Ingenuity software (www.ingenuity.com). A Core Analysis was performed to identify their involvement in biological processes, pathways and molecular networks.

### Cell culture

The breast lines used in this study were purchased from the American Type Culture Collection (ATCC) and cultured and maintained as per ATCC recommendations and as described previously^55^. The MDA-MB231LM2 RARRES3-overexpressing cell line was obtained from Roger Gomis and cultured as previously described^16^. All the cell lines were regularly tested for mycoplasma infection and authenticated using short tandem repeat profiling by scientific services at QIMR Berghofer Medical Research Institute.

### Reverse transcriptase –quantitative PCR

RNA was extracted using RNEasy Mini Kit (Qiagen, Venlo, Limburg, Netherlands) and cDNA synthesized using the SuperScript III First-Strand Synthesis System (Life Technologies) according to manufacturer’s instructions. RT-qPCR was performed on a LightCycler 480 (Roche, Basel, Switzerland) using SYBR Green (Roche) and normalised against β-actin or HRPT1 as an internal control. The following primers were used:

***RARRES3*** FOR–5′-GATCCATCTGGCTCCTCCAAG-′3, REV – 5′-GCTGTTGTTGACCCGATAGC-3′

***PSMB8*** FOR – 5′-GTCCAACATGATGTGCCAGT-3′, REV – 5′-ATTTCCTGAGAGCCGAGTCC-3′

***PSMB9*** FOR – 5′-ACTGTGCACTCTCTGGTTCAG-3′, REV – 5′-CCAGCCAGCTACCATGAGAT-3′

***PSMB10*** FOR – 5′-CTAGAAGACCGGTTCCAGCC-3′, REV – 5′-TGTGGTTCCAGGCACAAAGT-3′

***TAP1*** FOR – 5′-TTCGTGGGTGACGGGATCTA-3′, REV – 5′-GACGTGTCCTCTGTTACCCG-3′

***TAP2*** FOR – 5′-GGACCTCGGTTTCTTCCAGG-3′, REV – 5′-GGGTGAGTCGAGGCGATATG-3′

***IRF1*** FOR – 5′-CCCTGGCTAGAGATGCAGATT-3′, REV – 5′-TATCGGCCTGTGTGAATGGC-3′

***PSMB5*** FOR – 5′-AGGTCATAGGAATAGCCCCG-3′, REV – 5′-TGTGGCTGGGATAAGAGAGG-3′

***PSMB6*** FOR – 5′-TGGTAGGTGACAGCATCAGC-3′, REV – 5′-CGGACTCCAGAACAACCACT-3′

***RORA***FOR – 5′-ACATGTGAAGGCTGCAAGGG-3′, REV- 5′-GTTGGCAGCGGTTTCTACTG-3′

***Β-ACTIN***FOR – 5′-CCCAGAGCAAGAGAGAGG-3′, REV – 5′-GTCCAGACGCAGGATG-3′

***HPRT1*** FOR – 5′-CCTGGCGTCGTGATTAGTGAT-3′, REV – 5′-AGACGTTCAGTCCTGTCCATAA-3′

### Small interfering RNA (siRNA) transfection

Indicated cell lines were reverse transfected using either 10 nM individual or pooled siRNAs for 24–48 hours using Lipofectamine RNAiMAX as previoulsyd described^55^. The following siRNAs were used:

siRARRES3_2: 5′-CAAGACUGAAGGAUCAAUAAACAGC-3′

siRARRES3_3: 5′-CAGCUUGGACCAUGAGUACCAACCA-3′

siRARRES3_4: 5′-CCUCCAGUGUCUUCUCAGUCCUGAG-3′

siRORA_1: 5′-CAGGAAUCCAUUAUGGUGUCAUUAC-3′

siRORA_1: 5′-CUAGAAUGUCUGAAGUACAAACATG-3′

siIRF1_1: 5′-UGCAGAUUAAUUCCAACCAAAUCCC-3′

siIRF1_2: 5′-AGCGCCUUGGUAUGACUUAAAAUTG-3′

Scramble siRNA: 5′-UUCUCCGAACGUGUCACGUTT-3′

### Immunoblotting

Immunoblotting was performed as described earlier^56^. The Super Signal chemiluminescent ECL-plus (Amersham) was used for IP and CP-related proteins detection. The amounts of indicated proteins and loading-control COX-IV were measured by quantifying the band intensities by ImageJ software. Relative fold change was calculated using scramble siRNA transfected cells.

### Statistical analysis

Student’s *t*-tests, one-way analysis of variance (ANOVA) with Bonferroni post-hoc testing was performed using GraphPad Prism v6.0 (GraphPad Software, LaJolla, CA, USA) and the *p*-values were calculated as indicated in figure legends. Asterisks indicate significant differences (*p < 0.05; **p < 0.01; ***p < 0.001; ****p < 0.0001), n.s. = not significant.

## Additional Information

**How to cite this article**: Anderson, A. M. *et al*. The metastasis suppressor RARRES3 as an endogenous inhibitor of the immunoproteasome expression in breast cancer cells. *Sci. Rep.* **7**, 39873; doi: 10.1038/srep39873 (2017).

**Publisher's note:** Springer Nature remains neutral with regard to jurisdictional claims in published maps and institutional affiliations.

## Supplementary Material

Supplementary Figures

Supplementary Tables
