# Supplementary material for: The metastasis suppressor RARRES3 as an endogenous inhibitor of the immunoproteasome expression in breast cancer cells
Source: Sci Rep. 2017 Jan 4;7:39873. doi: 10.1038/srep39873 (PMC5209724; doi:10.1038/srep39873)
Supplement: Supplementary Figures [file srep39873-s1.pdf]

The metastasis suppressor RARRES3 as an endogenous inhibitor of the immunoproteasome expression in breast cancer cells

Alison M. Anderson, Murugan Kalimutho, Sarah Harten, Devathri M Nanayakkara, Kum Kum Khanna and Mark A. Ragan

A.

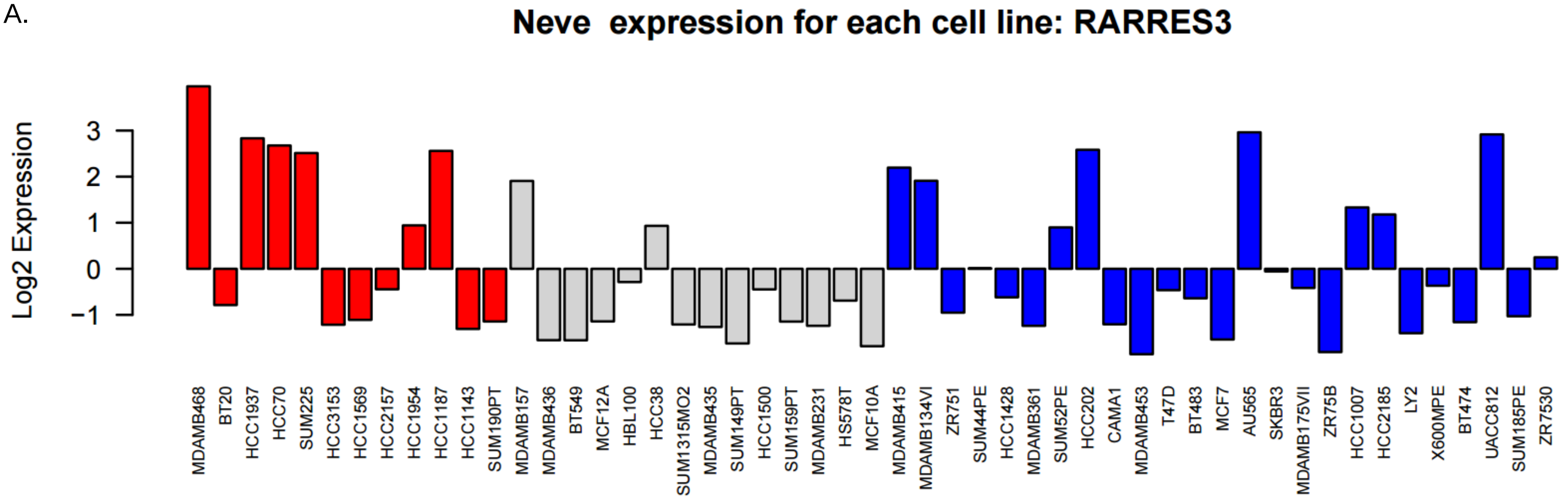

B.

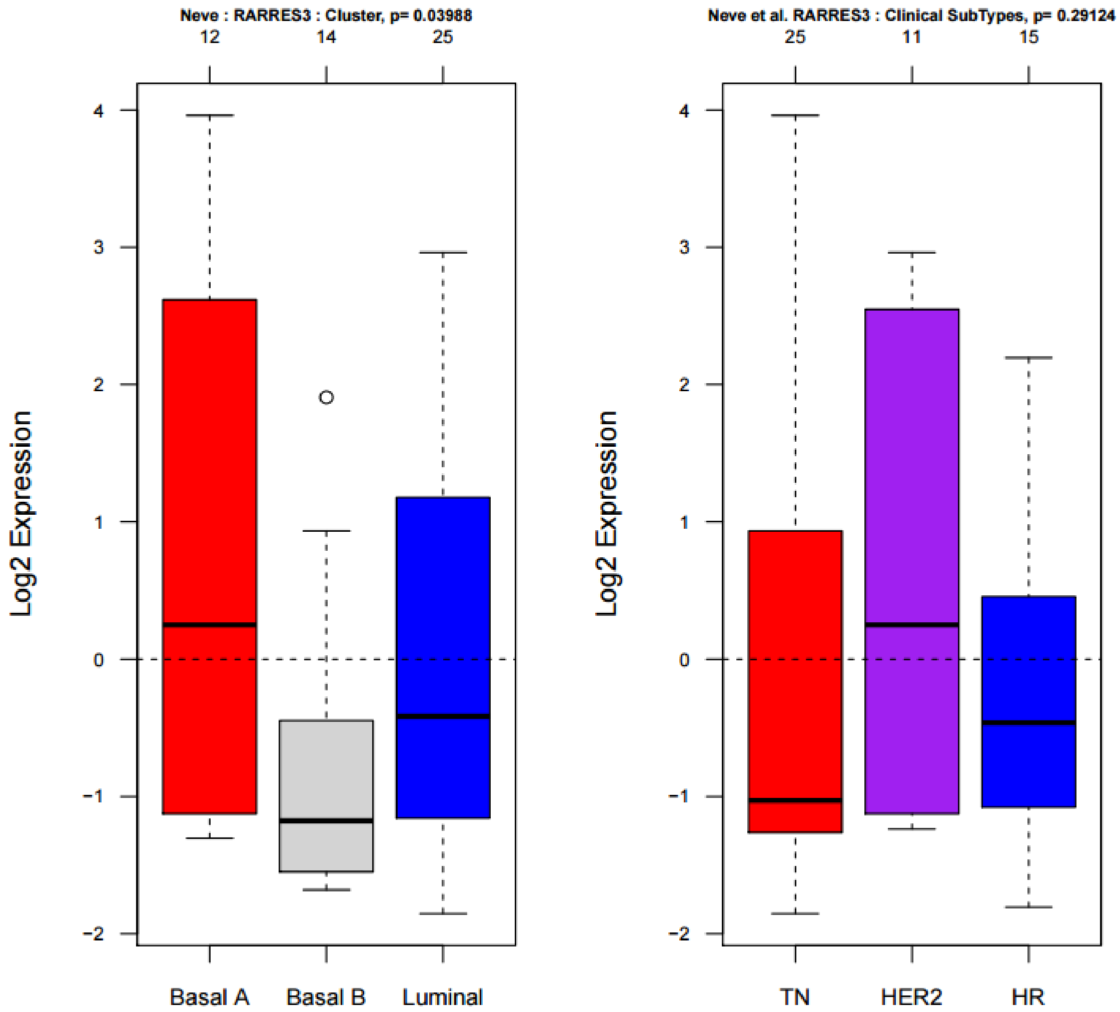

Supplementary Figure 1: (A) Log 2 *RARRES3* messenger RNA expression in a panel of breast cancer cell lines according to Neve et al. 2006 obtained through GOBO software (http://co.bmc.lu.se/gobo/). (**B**). Log 2 *RARRES3* messenger RNA expression between subtypes with indicated p value. TN: Triple negative; HR: hormone receptor

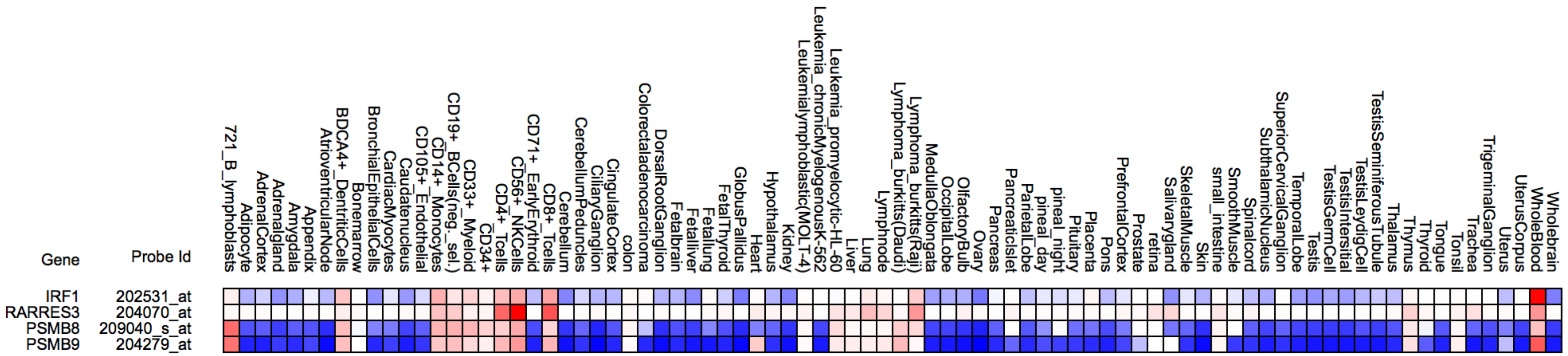

Supplementary Figure 2: Basal expression analysis across various human tissues and cells. The heat map shows the expression of *IRF1*, *RARRES3* and the immunoproteasome subunits *PSMB8* and *PSMB9* in their resting, unstimulated state across various tissues and cells. Data obtained from the Interferon database (http://www.interferome.org/)
